# Role of CT Perfusion in Monitoring and Prediction of Response to Therapy of Head and Neck Squamous Cell Carcinoma

**DOI:** 10.1155/2014/917150

**Published:** 2014-07-21

**Authors:** Lorenzo Preda, Sonia Francesca Calloni, Marco Elvio Manlio Moscatelli, Maria Cossu Rocca, Massimo Bellomi

**Affiliations:** ^1^Division of Radiology, European Institute of Oncology, University of Milan, Via Ripamonti 435, 20141 Milan, Italy; ^2^University of Milan, Via Festa del Perdono 7, 20122 Milan, Italy; ^3^Division of Medical Oncology, European Institute of Oncology, Via Ripamonti 435, 20141 Milan, Italy

## Abstract

This review aims to summarize the technique and clinical applications of CT perfusion (CTp) of head and neck cancer. The most common pathologic type (90%) of head and neck cancer is squamous cell carcinoma (HNSCC): its diagnostic workup relies on CT and MRI, as they provide an accurate staging for the disease by determining tumour volume, assessing its extension, and detecting of lymph node metastases. Compared with conventional CT and MRI, CTp allows for obtaining measures of tumour vascular physiology and functional behaviour, and it has been demonstrated to be a feasible and useful tool in predicting local outcomes in patients undergoing radiation therapy and chemotherapy and may help monitor both treatments.

## 1. Introduction

Head and neck cancers represent about 5% of all malignancies newly diagnosed each year. Squamous cell carcinoma is the most common histology, accounting for about 90% of these tumours. Head and neck squamous cell carcinomas (HNSCC) arise from the mucosa of the upper aerodigestive tract and are linked by common characteristics including a male-predominant presentation and a multifactorial etiopathogenesis. Historically tobacco and alcohol assumption are the most important risk factors while human papilloma virus (HPV) exposure is an emerging cause, particularly common in the oropharynx subsite and with a better clinical outcome [1].

Most head and neck patients present in a locally advanced stage with a poor prognosis. In this setting various strategies have been tried to improve outcomes of the two main standard treatments (surgery and radiotherapy). Concomitant chemoradiation treatment has become the standard of care in the unresectable locally advanced disease and as organ preservation strategy [2]. Induction polychemotherapy (given before radiotherapy with or without concomitant chemotherapy) has been extensively investigated on the effort of improving overall survival by reducing the incidence of distant metastasis [3–5]. Despite the wide literature on this topic, this approach cannot be considered a standard of care yet and needs further data. Finally the overexpression of epidermal growth factor receptor in HNSCC is more than 90% and a correlation between this feature and a worse prognosis was found. Cetuximab, a monoclonal antibody against epidermal growth factor receptor, showed significant efficacy in locoregional control of disease and in overall survival either in the curative setting [6] or in the recurrent/metastatic HNSCC [7].

Given all these new therapeutic approaches, there remains the fact that a subset of patients obtain a major or complete response, especially from induction chemotherapy and target therapy, and we do not have predictive markers to anticipate this and to personalize the therapeutic strategy in order to improve outcomes or reduce toxicity.

In the clinical practice cross-sectional imaging integrates endoscopic evaluation of HNSCC providing information about the local invasion of the tumour into the surrounding structures as well as the regional spread of the disease, as both have an impact on treatment and prognosis.

The traditional evaluation of response to treatment is based on modification of tumour dimensions which is unidimensional for the universally recognized Response Evaluation Criteria in Solid Tumour (RECIST) [17].

The assessment of tumour volume changes after treatment by CT may be used as an objective and reproducible technique for therapy monitoring, with good correlation with histology [18].

Furthermore CT-determined tumor volume is a strong predictor of local and locoregional outcome of laryngeal carcinoma [19].

However cross-sectional imaging techniques provide only morphologic assessment and do not tell us anything about the tumour biology.

The knowledge about the cellularity or the perfusion of a tumour may help in the differentiation of the biological behaviour during and after treatment of lesions having the same histologic type [20].

CT perfusion (CTp) has recently been used to obtain measures of tumour vascular physiology and hemodynamic. In contrast to the logarithmic relation between signal intensity and concentration of paramagnetic contrast medium of dynamic contrast enhancement MRI (DCE-MRI), the main advantage of CTp is the linear relationship between contrast concentration and attenuation in CT, which facilitates quantitative measurement of perfusion parameters [21]. Also, CTp advantages include high spatial resolution and wide availability, having the use of ionizing radiation, need of iodinated contrast medium injection, and relatively limited coverage as its major limitations.

## 2. CTp Technique

CTp is a theoretical tool able to quantify, through mathematical models and dedicated software, the “real” perfusion of tissues. The first technical requirement is the execution of repeated CT scans of the volume being analysed during and after intravenous administration of a fast bolus of iodinated contrast medium, to allow the study of the density variations over time [22]. The density measured by CT in the unit of volume (voxel), expressed in Hounsfield units (HU), reflects the contrast agent within the blood vessels and the contrast agent which has moved to the interstitial space due to passive diffusion [23].

The selection of the arterial input through the placement of a region of interest (ROI) on an artery allows obtaining a time-density curve of the artery, expressed in HU. This is then compared with the time-density curve of the tissue being analysed in order to distinguish between the quantity of contrast agent within the blood vessels (vascular compartment) and the quantity present in the interstitium (extra vascular/extra cellular compartment) [23].

Some studies demonstrated that the use of internal carotid artery (ICA) or external carotid artery (ECA) ipsilateral or contralateral to the tumour as the arterial input has no significant effect on CTp calculation of HNSCC [24, 25]. Tawfik et al. recommend the use of ICA because of the lower risk of partial volume effects correlated with its larger caliber and its course, almost perpendicular to the axial plane [24].

Various kinetic models can be used to calculate the distribution of contrast agent in the different compartments through the estimation of the perfusion parameters. In some of these models the analysis is performed using the single-compartment or double-compartment method which respectively describes the vascular and extravascular compartments as single or separate compartments [26].

The deconvolution method uses arterial and tissue time-density curves to calculate the impulse residue function (IRF), a theoretic tissue curve obtained assuming that the contrast agent is not diffusible and its concentration in the tissue is linearly dependent on the input arterial concentration when the blood flow is constant [26].

Most papers published about CTp in the study of HNSCC used the deconvolution based software which generates the following perfusion parameters [23, 27].

- *Blood flow* (BF), expressed in mL/min/100 g of tissue, is the flow rate of blood through the vasculature in tissue region. BF includes flow information from large vessels, arterioles, capillaries, and venules as well as arteriovenous shunts, which are more common in neoplastic tissue than in healthy tissue.
- *Blood volume* (BV), expressed in mL/100 g of tissue, represents the volume of blood that flows within vasculature in a tissue region.
- *Mean transit time* (MTT), expressed in seconds, represents the mean time the blood takes to pass through the microvasculature from the arterial to the venous end. MTT is inversely correlated to BF.
- *Permeability-surface products* (PS), expressed in mL/min/100 g of tissue, measure the product between the permeability and the total surface area of capillary endothelium in a unit mass of tissue (usually 100 g of tissue). It is considered as a surrogate marker of immature leaky vessels which are more common in neoplastic tissue.

Clinical interpretation of CTp is based on a qualitative analysis and a quantitative analysis. Qualitative analysis involves the analysis of the colour maps generated by the software for each perfusion parameter. Each pixel of the CT images is attributed a colour which represents the numerical value of perfusion parameter calculated for that pixel and the colour scale is chosen by the operator in order to maximize the differences between areas having different perfusion.

Quantitative analysis involves the interpretation of numerical values of perfusion, which the software calculates for the area bounded by the ROI placed over the tumour representing the mean of the numerical values of each voxel included in the ROI [23].

Similar to CTp studies of other tumour sites [28], Petralia et al. found a good inter- and intraobserver agreement when CTp data were analysed by differently experienced readers, especially for BF, BV, and MTT [25]. The lower and more variable agreement observed for PS probably depends on the fact that its calculation, being reliant on prolonged scanning, is likely more affected by the cumulative effects of small motions than the other parameters which derive from first-pass scanning [25].

Over the past decade several studies investigated the role of CTp both in monitoring and predicting short-term and long-term response to organ-preserving treatments in HNSCC, though different end points were considered: disease-free survival in some case [16] and tumour volume reduction in others [9, 13, 14].

## 3. Monitoring during and after Treatment

In the clinical routine endoscopic examination integrated by biopsy and cross-sectional imaging represents the gold standard for therapy monitoring of HNSCC.

**Figure 1:**
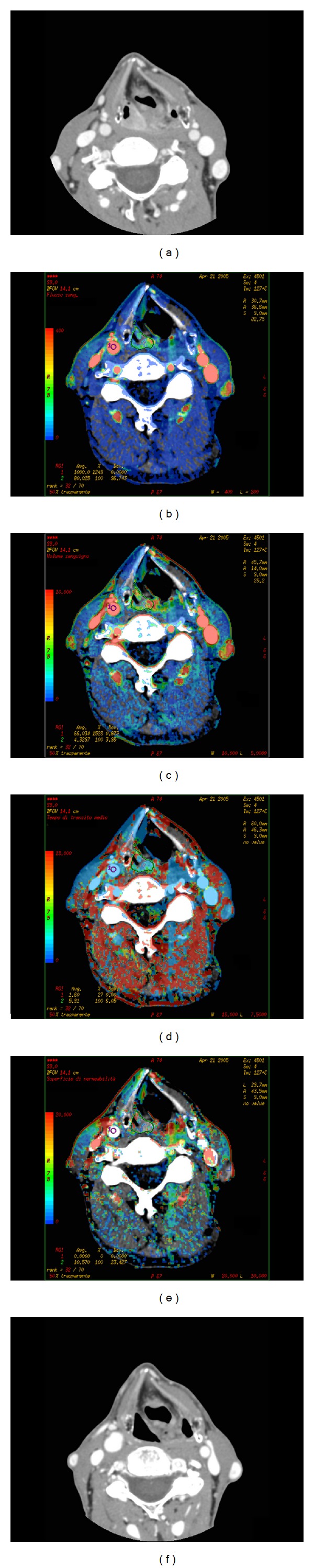
Squamous cell carcinoma of hypopharynx in a responder patient: CT scan (a) obtained before chemotherapy shows the lesion involving the right piriform sinus. On the same section, functional maps of BF (b), BV (c), MTT (d), and PS (e) are automatically generated by the software, showing the values calculated in each pixel of the image in a color scale. CT scan obtained in the same patient after chemotherapy and radiotherapy showing a complete disappearance of the tumour (f).

Few studies (Table 1) investigated the value of CT-determined tumour perfusion in this specific clinical setting in a similar way to what demonstrated for tumours located in other body regions. This is based on the theory that changes produced by radiotherapy and chemotherapeutic agents on tumour vascularity can be identified by changes in CTp measured tumour perfusion. Treatment, specifically, induced reduction of microvessels inside the tumour could be identified as a decrease of BV values while a decrease of BF could indicate a reduction of low resistance flow arteriovenous shunts in the microvasculature. The reduction of hyperpermeable capillary bed could be expressed with a decrease of PS values [29] (Figure 1).

In a small group of 9 patients treated with induction chemotherapy Gandhi et al. [10] found a positive correlation between BV pretreatment lesion values and the tumour response assessed by endoscopy. They also found that a decrease ≥ than 20% in BV values after 3 weeks of therapy is able to predict a reduction of cancer volume greater than 50%.

Petralia et al. [9] confirmed the relevance of CTp parameters in monitoring the response to the therapy. They found a significant correlation between the percent change in BV and BF values and the percent reduction of tumour volume after 3 cycles of induction chemotherapy. Their results appear to be more reliable since they use, as response quantification, tumour volume assessed by CT instead of the endoscopy standard which is an operator-dependent technique.

Serial fluctuations in perfusion parameters of HNSCC during a course or radiotherapy were prospectively evaluated by Truong et al. [8], by using CTp to provide information about the periodical changes in tumour oxygenation produced by radiotherapy. Pretreatment tumour BF was higher in patients who achieved locoregional control (LRC) compared with those with locoregional failure (LRF) (*P* = 0.004), consistent with the findings of Hermans et al. [12]. Furthermore, authors demonstrated that an increase in BF during the first two weeks of radiotherapy is able to predict LRC, with a decrease in both groups after 6 weeks of radiotherapy compared with the values at the baseline scanning, suggesting that a higher BF in tumour tissue at the baseline and during the early course or radiotherapy predicts a better tumour control.

Interestingly Šurlan-Popovič et al. [11], in a series of 24 patients with locally advanced HNSCC, demonstrated that the modifications of CTp parameters during the course of treatment might predict tumour response to cisplatin-based chemoradiotherapy.

In particular responders presented a significant reduction of BF values (*p* 0,04) after 40 Gy which was more pronounced after 70 Gy (*p* 0,01) and a significant reduction of BV values after 40 Gy with a plateau after 70 Gy (*p* 0,04). MTT and PS values showed nonsignificant modifications.

On the contrary in nonresponders BF, BV, and PS values showed a nonsignificant increase after 40 Gy.

The possible explanation given by the authors to these findings is that the dynamics in CTp parameters are mainly related to the cytotoxic effects of radiotherapy to the vascular endothelium which is more effective than the little antivascular action of cisplatin-based chemotherapy [11].

In an attempt to explain the increase of perfusion parameters values observed in nonresponders, authors hypothesized that ionizing radiation therapy may produce the upregulation of VEGF which promotes survival of endothelial cells in residual tumour tissue and consequently radiation resistance [30].

## 4. Prediction of Response to Radiotherapy and Chemotherapy

The rationale of the potential role of CTp in predicting response to nonsurgical therapies is that it is substantially influenced by tissue perfusion and local oxygen delivery. The oxygen supply to a tissue is governed by its perfusion and the arterial oxygen concentration. It is conceivable that higher levels of BF and BV may correlate with better oxygen/drug delivery and therefore may predict the response to radiation therapy or chemotherapy.

Theoretically, a CTp study performed prior to the beginning of therapy could identify patients with poorly perfused tumours likely to demonstrate a bad response to chemoradiotherapy, thus allowing them to be directed to alternative treatments [29].

Thus, in recent years, some studies appeared in the literature dealing with this topic (Table 2).

**Figure 2:**
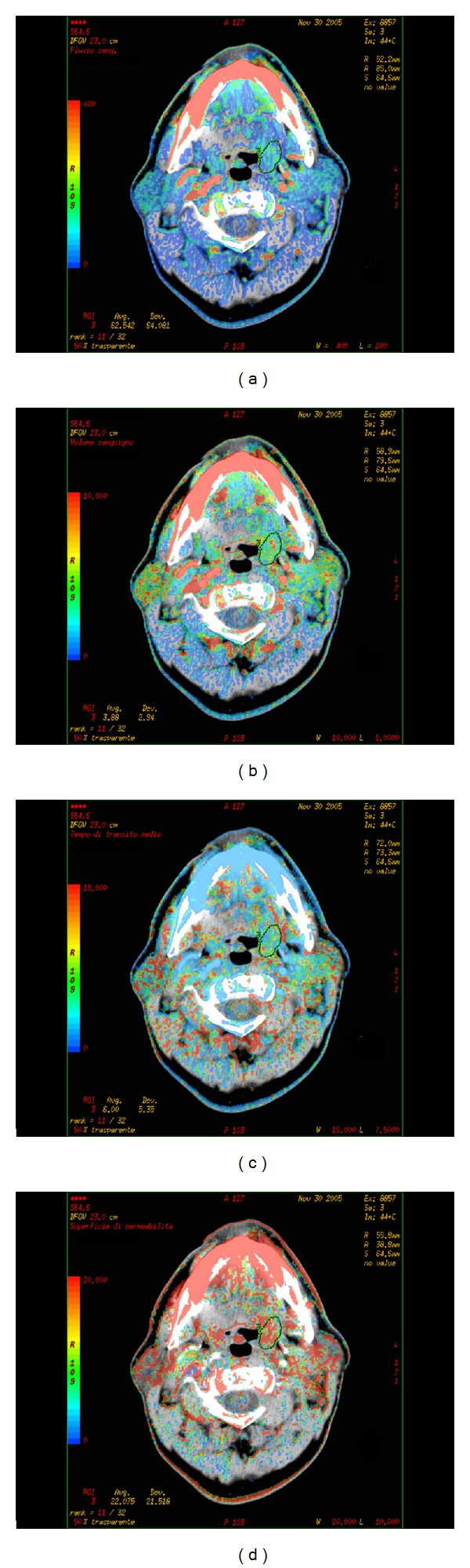
Squamous cell carcinoma of the oropharynx in a nonresponder patient. The functional maps of BF (a), BV (b), MTT (c), and PS (d) are automatically generated by the software, showing low BF and BV values within the lesion.

In a series of 105 patients with HNSCC treated with definitive radiotherapy, some associated with adjuvant chemotherapy, Hermans et al. [12] found that CTp parameters are independent predictors of local control together with T stage. In particular patients with a low perfusion pretreatment value showed a statistically significantly higher local failure rate than those with a high perfusion value (*P* < 0.05), presumably due to a more extensive degree of hypoxia in low-perfused tumour and therefore characterized by low radiosensitivity (Figure 2).

Similarly, in a study of 17 patients, Zima et al. [13] demonstrated that elevated pretreatment values of BF (*P* < 0.03) and BV (*P* < 0.004) CTp parameters showed a significant correlation with response to induction chemotherapy evaluated endoscopically.

Bisdas et al. [14] confirmed these results showing correlation between radiological response after induction chemotherapy and CTp parameters at baseline while just a weak correlation with pretreatment tumour volume was found. With these findings CTp perfusion parameters seemed to outperform the morphologic characteristics, being significantly different (*P* ≤ 0.002) between responders and nonresponders with high BF, BV, and PS and low MTT correlated with a better tumour response, presumably reflecting better tumour oxygenation. The authors concluded their paper highlighting the role of CTp as a noninvasive, inexpensive, and widely accessible diagnostic tool, able to improve the choice of organ preservation treatment regimens in order to maximize the therapeutic efficacy [14].

In the paper by Petralia et al. [9] only baseline tumour BV was significantly lower (*p*  0,015) in nonresponder compared to responders to induction chemotherapy. They found a trend to correlation between baseline tumour BV and high tumor volume reduction assessed by CT [21].

Bisdas et al. [15] assessed whether CTp may predict outcome in 21 chemoradiated patient with oral cavity, oropharynx, and hypopharynx SCC after surgical excision: they applied a new analysis on the region of interest-derived CTp values, namely, the maximum BF, BV, and PS, as well as the minimum MTT values, trying to avoid the considerable intratumoral variation covered with mean values. Both mean perfusion values and BF_max⁡_, BV_max⁡_, and BS_max⁡_ were significantly different between patients with and without tumour recurrence (*P* < 0.04). Also, the authors underline the predictive value of PS and MTT. In particular they found a relative risk of recurrence about 14 time higher in patients with lower than the median BS_mean_ values, which is apparently in contrast with the results of other studies demonstrating a significantly shorter disease-free survival in patients with high intratumoral microvessel density [31]. According to the authors these findings confirm the absence of any clear relationship between microvascular density and PS values [32]. On the other hand the predictive role of MTT values may be attributed in part to leaky tumour vessels, which lead to improved oxygenation [15].

The same authors [16], in a large series of 84 patients with advanced HNSCC who underwent CTp prior to concomitant chemoradiotherapy, found that BF and PS values were significantly higher in patients who had local failure (*P* ≤ 0.02).

Furthermore a simultaneous visual evaluation of the BF and the BV parametric maps showed that the presence of a mismatch (>30% of the examined lesion extent) between the colour-encoded maps correlates with shorter life expectance (*P* = 0.01) and smaller recurrence-free survival (*P* = 0.03).

This approach is based on the rational that a considerable mismatch, more evident in locally advanced tumours, may indicate a heterogeneous pattern of vascularisation, which may lead to a cascade that influences cellular phenotypes and presents with therapy resistance [33].

## 5. Conclusions

All the preliminary results of previous studies show that elevated CT perfusion parameters are statistically correlated to a better response to radiotherapy and chemotherapy and prove that tissue oxygenation may also influence the spread of chemotherapy agents and a higher radiosensitivity.

BV and BF have clearly emerged as the most significative CTp parameters as they may predict response to radiation therapy and chemotherapy and may help monitor both treatments.

PS and MTT parameters are insufficiently predictive of the response compared with BV and BF: only one paper highlighted their achievable effectiveness [15].

It must be also considered that published studies considered different end points and most of them correlated their findings with short-term follow-up.

Large-scale studies examining the long-term predictive value of baseline CTp studies in patient treated both with neoadjuvant chemoradiation or chemoradiation with curative intent might help in tailoring the therapy regimen on individual basis. As an instance, the possibility to identify earlier a failed response to induction chemotherapy may help avoid this step during the therapeutic treatment plan.

Therefore, a more standardized technique is hopeful in order to achieve reproducible and comparable method across different institutions.

Future integrated applications with PET-CT findings or dual-energy CT may enable more understanding of the biologic behaviour of the tumour in vivo, making the CTp one of the potential cornerstone of biologic imaging of HNSCC.

## Figures and Tables

**Table 1:** CTp parameters obtained during and after radiotherapy and induction chemotherapy showing as valid predictions in monitoring the treatment.

Author	Cancer	Treatment	Number of patients	Predictive parameters	*P* value	Type of study
Truong et al. [8]	Primary HNSCC	Radiotherapy	15	BF	0.046	Prospective
				BV	0.053	

Petralia et al. [9]	SCCA of the upper aerodigestive tract	Induction chemotherapy		BF	<0.003	Prospective
			25	BV	<0.01	

Gandhi et al. [10]	SCCA of the upper aerodigestive tract	Induction chemotherapy	9	BV	—	Prospective

Šurlan-Popovič et al. [11]	Advanced SCCA of oral cavity, oropharynx, hypopharynx	Chemoradiotherapy	20	BV	0.01	Prospective

Notes: SCCA: squamous cell carcinoma; HNSCC: head and neck squamous cell carcinoma; BF: blood flow; and BV: blood volume.

**Table 2:** Results for the prediction of response to radiotherapy and chemotherapy based on the pretreatment tumour volume and the perfusion-associated parameters.

Author	Cancer	Number of patients	Predictive parameters	*P* value	Type of study
Hermans et al. [12]	Primary HNSCC	105	Median perfusion value	0.01	Prospective

Zima et al. [13]	SCCA of the upper aerodigestive tract	17	BF	<0.03	Prospective
			BV	<0.004	

Bisdas et al. [14]	Advanced oropharynx SCCA	19	BF, BV, PS, MTT	<0.001	Prospective

Petralia et al. [9]	SCCA of the upper aerodigestive tract	25	BV	0.015	

Bisdas et al. [15]	Primary SCCA of oral cavity, oropharynx, hypopharynx	21	BF, BV, MTT, PS	<0.004	Prospective
			BF_max_, BV_max_	<0.001	

Bisdas et al. [16]	SCCA of the upper aerodigestive tract	84	BF, PS	<0.000	Prospective
			BF-BV mismatch	0.01	

Notes: SCCA: squamous cell carcinoma; HNSCC: head and neck squamous cell carcinoma; BF: blood flow; BV: blood volume; PS: permeability surface area product; MTT: mean transit time; and CP: capillary permeability surface area product.
